# Saccadic Reaction Times and Computational Modelling Reveal Heterogeneous Binocular Summation in Glaucomatous Visual Fields

**DOI:** 10.1007/s44402-026-00100-9

**Published:** 2026-05-09

**Authors:** Ashwini Venkat Reddy Chanakya, Johan JM Pel, Ronnie George, Peter Bremen

**Affiliations:** 1 Department of Neuroscience, Erasmus MC, Rotterdam, The Netherlands; 2 Medical Research Foundation, Sankara Nethralaya, Chennai, India

**Keywords:** Binocular vision, Eye movements, Perimetry, Promptness, Statistical facilitation

## Abstract

Binocular summation enhances visual performance. This enhancement may be disrupted in visual conditions that introduce interocular asymmetries, e.g., glaucoma. Reaction times provide a direct index of visual processing efficiency. However, they have not been studied systematically under binocular conditions in glaucoma. This investigation quantified monocular and binocular saccadic reaction times (RTs) across the visual field using eye-movement perimetry (EMP), compared binocular summation between healthy and glaucoma participants and evaluated correspondence with computational models. Saccadic RTs were measured monocularly/binocularly at 54/56 visual-field locations in seven healthy participants and eight participants with glaucoma of varying severity. Each location was tested with six to ten repetitions under low (74%) and high (155%) contrast conditions. Data were analysed using reciprobit plots, estimation statistics and linear mixed-effects modelling. Binocular RTs were compared with statistical facilitation (Race model) and variance-weighted neural integration predictions. Healthy participants consistently demonstrated a binocular advantage, with RTs faster by ~20 ms relative to monocular viewing, closely matching statistical facilitation predictions. Glaucoma participants showed greater heterogeneity, ranging from preserved summation (~40 ms faster than monocular) to binocular RT being slower than the fastest monocular RT. Computational modelling indicated that most glaucoma cases were compatible with statistical facilitation, but some exhibited deviations suggestive of inhibitory interactions, monocular dominance or faster responses than predicted by statistical facilitation. Binocular RTs provide a sensitive index of visual processing and reveal heterogeneous binocular summation mechanisms in glaucoma. EMP under binocular conditions offers a promising approach for studying functional visual impairment beyond traditional monocular testing.

Key Points

- Binocular summation normally speeds up visual processing, but can be reduced or absent in glaucoma. Glaucomatous saccadic reaction times reveal heterogeneity, including reduced summation, better-eye dominance or inhibition.
- In healthy observers, binocular reaction times follow statistical facilitation predictions. In glaucoma, modelling shows diverse patterns, that range from facilitation to slower, faster or inhibitory interactions.
- Binocular and monocular eye-movement perimetry quantifies binocular reaction-time benefits and their loss in glaucoma, providing functional information not captured by traditional monocular clinical tests.

## Introduction

Binocular vision is fundamental to human visual performance. The integration of inputs from the two eyes creates a unified percept that supports stereopsis, spatial orientation and interaction with the environment [1–4]. A defining feature of binocular vision is binocular summation, whereby binocular performance exceeds that of monocular performance [2, 3]. This phenomenon is a well-documented characteristic of the visual system [1, 5–10]. At the neural level, summation is mediated by the convergence of monocular inputs onto binocular neurons, which increases signal strength and reduces variability [11–13]. Functionally, this results in enhanced contrast sensitivity [14], faster reaction times [14] and more stable performance under visually demanding conditions [15].

This study focuses on binocular and monocular reaction times using goal-directed eye movements, namely saccades [16]. Reaction times provide a sensitive marker of visual processing efficiency, reflecting the speed at which sensory input is transformed into motor output. Unlike threshold-based measures, reaction times reflect the dynamics of information processing, which are critical for everyday activities requiring rapid responses. Several computational models have been proposed to explain binocular reaction times. The Race model assumes statistical facilitation, in which each eye processes the stimulus independently and the faster signal elicits the response [17, 18]. However, binocular reaction times can exceed statistical-facilitation predictions, indicating additional neural integration [18, 19]. The Linear Approach to threshold with Ergodic Rate (LATER) model can account for the faster-than-predicted binocular response [20, 21].

This model conceptualises saccadic initiation as a linear accumulation of sensory evidence toward a decision threshold. Binocular inputs accelerate accumulation and thereby shorten reaction times. The combination of the two monocular inputs in the LATER model can be achieved within a Bayesian cue integration framework, which proposes that binocular inputs are weighted according to their reliability [22–24]. Strong summation is expected when both eyes provide reliable information, whereas reduced summation or inhibition occurs when one eye is degraded. Together, the statistical-facilitation and the neural-integration models provide benchmarks for comparison with empirically observed data, particularly in clinical conditions, in which interocular asymmetry in visual acuity [25–27], ocular alignment [26] and contrast [27, 28] is common.

Despite the importance of binocular vision, most clinical assessments are typically performed under monocular conditions [29]. While monocular testing provides useful diagnostic information, it does not always capture binocular interactions that determine visual performance during natural viewing [30–34]. Glaucoma is one condition in which the binocular integration mechanism may be altered. It is characterised by progressive loss of retinal ganglion cells, resulting in visual-field defects that often differ between the two eyes [27, 34–36]. This interocular asymmetry can reduce the effectiveness of binocular summation and, in some cases, lead to binocular inhibition, e.g., binocular contrast sensitivity is worse than that of the better eye alone [14, 37, 38]. Previous studies using contrast sensitivity and threshold-based measures have demonstrated impaired binocular integration in glaucoma [27, 31, 32, 38]. However, threshold-based measures do not capture the temporal dynamics of visual processing.

Reaction time provides a complementary measure of processing speed and sensorimotor transformation. Eye movement perimetry (EMP) has been used to measure monocular saccadic reaction times across the visual field and has demonstrated delayed responses in glaucoma [36, 39, 40]. These findings highlight the sensitivity of reaction-time metrics to functional impairment. Despite this, EMP has been applied solely under monocular conditions, and the effects of binocular viewing on reaction times in glaucoma remain insufficiently characterised.

The present study applies EMP under binocular conditions to characterise reaction times and to evaluate their mechanistic basis with theoretical models. The objectives were to: (1) quantify the binocular reaction-time advantage relative to monocular viewing; (2) compare binocular reaction times between healthy and glaucoma participants, thereby evaluating differences in integration patterns and (3) compare empirically measured binocular reaction times to computational frameworks, such as statistical facilitation and neural integration. To enable location-specific comparisons, monocular (left, right) and binocular reaction times were measured at 54/56 spatial locations with low and high-contrast targets and multiple repetitions per condition. This detailed measurement protocol was necessarily time-intensive. Therefore, the study was conducted in a relatively small cohort of seven healthy and eight glaucoma participants spanning a range of disease severities. The goal was not a large-scale clinical evaluation but rather a detailed characterisation of binocular summation in glaucoma and to establish a methodological framework for future studies.

## Methods

### Participants

Fifteen individuals participated in the study, including eight females, two individuals of South-Asian descent (participants P2 and P15) and 13 Caucasians, with ages ranging from 24 to 67 years (see Table 1). Seven participants had a clinical diagnosis of glaucoma. Participant 11, the sibling of participant 10, was suspected of glaucoma due to optic disc changes and had a positive family history. None of the participants had ocular conditions (e.g., ptosis, corneal opacities or oculomotor nerve palsy) that could compromise the reliability of eye-tracking. All participants had spherical ametropia ≤ ±5.00 D, cylindrical ametropia ≤ 2.00 D and a best-corrected visual acuity ≤ 0.30 logMAR for distance and at least N8 (0.40 logMAR) for near tasks. Four glaucoma participants (P10, P12, P13 and P14) had previously undergone cataract surgery and were fitted with artificial intraocular lenses in one or both eyes. The sample included familial relatives and participants P2, P3 and P5 are authors of this study.

**Table 1:** Demographic and clinical characteristics of participants.

Participant ID	Age (years)	Gender	Clinical classification	Ophthalmic & functional presentation
1	24	F	Healthy participant	Normal visual function
2	31	F	Healthy participant	Normal visual function (Author); HFA_MD OD: 0.84 dB; OS: 0.02 dB
3	43	M	Healthy participant	OU: Myopic astigmatism with normal visual function (Author); HFA_MD OD: 0.59 dB; OS: 1.59 dB
4	45	F	Healthy participant	Normal visual function; Positive family history
5	50	M	Healthy participant	Normal visual function (Author); HFA_MD OD: 0.73 dB; OS: 1.52 dB
6	61	M	Healthy participant	Normal visual function
7	64	F	Healthy participant	OU: Healthy optic discs; Myopia OD = –3.50 D, OS = –5.00 D (no amblyopia); Positive family history (Sister of P13 & P14)
8	26	M	Glaucoma (Early)	OD: Focal visual field defect (enlarged blind spot); Positive family history
15	41	M	Secondary glaucoma	OS: Unilateral blunt trauma; superior arcuate field defect; no traumatic optic neuropathy; normal colour vision; HFA_MD OD: –3.18 dB; OS: –9.48 dB
9	61	F	Glaucoma (moderate)	OD: Focal visual field defect
10	63	M	Glaucoma (moderate)	OD: Inferior and nasal defects; OS: Early superior arcuate defect; HFA_MD OD: –4.29 dB; OS: –5.11 dB; Positive family history (Brother of P11)
11	65	M	Glaucoma suspect	OU: Glaucomatous optic disc changes; focal defects (enlarged blind spots); Positive family history (Brother of P10)
12	65	F	Glaucoma	OU: Nasal defects
13	66	F	Glaucoma	OU: Superior and nasal field deficits; Positive family history (Sister of P7 & P14)
14	67	F	Glaucoma (advanced)	OD: Dense superior arcuate field defect; OS: Early superior arcuate defect; HFA_MD OD: –13.29 dB; OS: –2.87 dB; Positive family history (Sister of P7 & P13)

*F* female, *HFA_MD* Humphery field analyser mean deviation (decibel, dB), *M* male, *OD* right eye, *OS* left eye, *OU* both eyes.

### Experimental Setup

A haploscopic display system was used to present visual stimuli to each eye independently (for a detailed description, see [41]). Two thin-film transistor (TFT) stimulus monitors projected images via front-surface and dichroic mirrors, forming a virtual binocular screen. An infra-red eye tracker with a sampling frequency of 120 Hz (Tobii Pro X3-120, Tobii, *tobii.com*) was positioned behind the dichroic mirrors, enabling unobstructed recording of both eyes. Participants were seated with their head stabilised using a chin rest and forehead support, and wore spectacle frames with opaque side blinders to minimise stray light.

Participant 15 was tested at our facility in India. All other participants were tested in a duplicate setup in the Netherlands. Measurement equivalence between sites was verified by testing three study authors at both locations, showing no significant differences in their reaction times across the haploscopic systems.

### Visual Stimuli and Target Locations

The stimuli were identical to those used in a previous study from our laboratory [41]. Briefly, they consisted of a green fixation circle (0.88° radius) and an achromatic target circle (0.44° radius) presented against a uniform grey background (6.2 cd/m²). Two Weber contrast (WC) levels were tested, 74% and 155%.

**Fig. 1:**
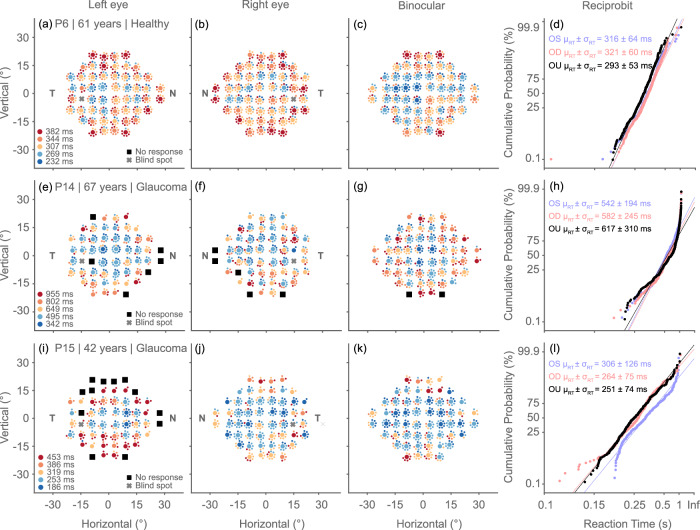
Saccadic reaction-time maps (**a**–**c**, **e**–**g**, **i**–**k**) and reciprobit plots (**d**, **h**, **l**) for a healthy participant (P6; **a**–**d**) and two glaucoma participants (P14, P15; **e**–**l**) tested at low contrast (74%). Circles are colour-coded by reaction time (blue = faster, red = slower), with large circles indicating median reaction times across repetitions and small, surrounding circles showing reaction times of individual repetitions. A complete ring of small circles denotes that goal-directed saccades were made for all repetitions (100%). The circle starts at 12 o’clock and runs clockwise. For instance, 3 o’clock indicates that 25% of the repetitions elicited a goal-directed response. Black squares denote locations with no valid saccade and the grey “X” marks the blind spot. The reciprobit plots (**d**, **h**, **l**) display cumulative probability distributions of promptness for left eye (OS, blue), right eye (OD, red) and binocular (OU, black) viewing, with linear fits (dashed lines) of the 25th and 75th percentiles. The text insets indicate the reaction time mean and standard deviation based on the fit. Note that the colour map range for each heat map is tailored to the individual participant’s specific reaction-time distribution. This individual scaling is utilised to maximise the visual resolution of localised functional patterns and spatial inhomogeneities within each visual field, which would otherwise be obscured or compressed by a uniform global scale.

Target locations were primarily expressed in polar coordinates, defined by angular direction (Φ) and radial eccentricity (*R*), spanning a range from 2.5° to 27°. This representation reflects the angular organisation of the oculomotor system (e.g., the superior colliculus), facilitating a systematic analysis by pooling reaction times along eccentricity [42]. The experimental grid was derived from the standard 24-2 Cartesian layout, but locations were shifted slightly to create their closest iso-eccentricity matches to simplify the analytical framework [41]. While analysis was performed in the polar domain, target locations are presented in Cartesian coordinates for visualisation (Fig. 1), following the convention that positive *x*-values represent the right visual field and positive *y*-values represent the superior visual field.

Monocular testing (both right (OD) and left (OS) eyes) followed the standard 24-2 grid with 54-locations. In this layout, two unique nasal locations at 27° eccentricity do not have corresponding temporal counterparts, resulting in a slight spatial asymmetry. To create a spatially symmetric 56-location grid for visualisation purposes, two geometrically corresponding temporal locations (at ±27°/±3°) were added to the binocular (OU) grid to complement the two nasal locations already present in the 24-2 layout. These additional temporal sites were utilised only to ensure symmetry in visualisation and were not included in the derivation of monocular-based model predictions. The monocular grids account for the anatomical limits of binocular vision and exclude the temporal-most locations (±27°/±3°), where interocular overlap is limited by the nasal bridge. The OU grid includes these locations to provide a complete and symmetric visualisation of the functional field. This ensured that reliability estimates and model predictions were derived exclusively from spatially corresponding locations between the two eyes.

### Behavioural Paradigm

The behavioural paradigm was identical to that described in our previous study [41]. Monocular responses from this dataset have been partially reported but were not analysed in detail, whereas the binocular data have not been reported and form the focus of the present study. All testing took place in a dimly lit, quiet room. Participants completed a block-randomised experiment that included six viewing conditions: monocular viewing of the left or right eye and binocular viewing, each tested at two WC levels (74% and 155%). Each block consisted of 54 and 56 locations under monocular and binocular viewing, respectively, presented in random order and repeated 2–4 times. Across all conditions, participants completed between six and 14 repetitions per location. However, for P13, a minimum of two (WC 155%: right and left eyes, WC 155%) and a maximum of five repetitions (all other conditions) were obtained. Here, ‘repetition’ refers to the number of valid trials recorded per target location and viewing condition. For each participant, individual blocks lasted 4–10 min and the total testing time was ~4 h, spread across multiple sessions over 3 months. Each session typically lasted 1–3 h, with regular short breaks and occasional longer breaks for comfort.

Before each block, participants underwent eye-tracker calibration using a nine-point grid that covered central and peripheral regions of the virtual display. Calibration ensured accurate conversion of eye position signals into visual angles, accounting for screen size and viewing distance.

Each trial began with a green fixation circle, which remained visible after target onset in accordance with the overlap paradigm commonly used in eye-movement perimetry [39]. After a random delay (10th/50th/90th percentile: 1404/1422/1460 ms), an achromatic target appeared at a randomly selected location. Participants were instructed to execute a rapid and accurate saccade toward the target. A closed-loop detection algorithm terminated the trial once five consecutive gaze samples fell within a radius of 5° around the target location, ensuring that the trial ended as soon as a valid saccade was recorded. If no response was detected, the trial ended after 1.2 s. The fixation circle was then relocated to one of five randomly chosen positions and the next trial commenced.

Before formal data collection, participants completed a training block consisting of 56 locations (one repetition, high contrast, binocular viewing). This familiarised them with the task and allowed verification of stable fixation and accurate goal-directed saccades. Based on feedback during training, the inter-trial speed of the fixation stimulus was adjusted individually to enhance comfort.

### Data Analysis

#### Extraction of Saccade Parameters

Calibrated eye-tracking data were processed as described previously [41] using custom-written MATLAB programs (R2024a, MathWorks Inc., *mathworks.com*). Traces from the right and left eyes were averaged and resampled to 1 kHz using modified Akima cubic Hermite interpolation (‘*makima’* in MATLAB’s ‘*interp1’* function), which preserves continuity and provides stable derivatives for velocity estimation. Blink detection was based on the velocity trace (Euclidean norm of horizontal and vertical derivatives) with a velocity threshold of 600°/s. Transitions around blink events were smoothed with a three-sample running average and signal loss was interpolated between the onset and offset of the blink.

Saccades were detected from the velocity trace using MATLAB’s ‘*findpeaks’* function with a minimum peak distance of 200 ms. A two-step velocity criterion was applied: candidate events were identified at 100°/s and onset/offset were refined using a 50°/s threshold. All trials were visually inspected and manually corrected if necessary (healthy: ~1%; glaucoma: ~28%, predominantly due to brief signal interruptions rather than systematic data loss).

For each detected saccade, onset and offset times as well as horizontal and vertical positions were determined. Saccadic reaction time, defined as the interval between target onset and saccade onset, is referred to as *reaction time* throughout this paper. Analysis included only goal-directed primary saccades that met the following criteria: (1) the first saccade after stimulus onset, (2) a reaction time >100 ms to exclude anticipatory responses and (3) spatial accuracy verified using a circular region of interest (ROI; 5° radius) centred on both the fixation and target locations. Gaze position was required to remain within the fixation ROI at stimulus onset (evaluated within a ±10 ms window) and the saccadic endpoint was required to fall within the target ROI at saccadic offset. This ROI-based criterion allowed for normal oculomotor variability while ensuring accurate identification of stimulus-directed responses.

#### Reaction-time parameter estimation

Reaction-time parameter estimation followed the approach reported previously by our laboratory [41]. Briefly, reaction times (RTs) were transformed into their reciprocals (promptness, *P* = 1/RT), which yields approximately normal distributions suitable for parametric analysis [20, 21, 43]. Promptness values per stimulus location were plotted against their cumulative probability in probit units, generating reciprobit plots [20]. A linear fit between the 25th and 75th percentiles provided two parameters: the mean promptness (*μ*_P_), representing average visuomotor speed and the standard deviation of promptness (*σ*_P_), reflecting variability. For interpretability, *μ*_P_ was expressed as its reciprocal (1/*μ*_P_) to approximate mean RT (*μ*_RT_), and *σ*_P_ was converted to an estimate of RT variability (*σ*_RT_) as

1 $${\sigma }_{{{RT}}}\approx \,\frac{{\sigma }_{{{P}}}}{{\mu }_{{{P}}}^{2}}.$$

#### Binocular Models

To evaluate binocular reaction times, the observed data were compared with predictions from a standard statistical facilitation (Race) model and a neural integration model based on variance-weighting. The statistical facilitation model assumes that the two eyes operate independently, and that the response is triggered by the faster of the two monocular signals [17, 18]. For two independent reaction time distributions, the cumulative probability of a binocular response occurring before time *t* is given by

2 $${F}_{{{{\rm{OU}}}}}(t,x)={F}_{{{{\rm{OS}}}}}(t,x)+{F}_{{{{\rm{OD}}}}}(t,x){\textstyle {\mbox{-}}}{F}_{{{{\rm{OS}}}}}(t,x)\cdot {F}_{{{{\rm{OD}}}}}(t,x) < {F}_{{{{\rm{OS}}}}}(t,x)+{F}_{{{{\rm{OD}}}}}(t,x)$$

where *F*_OU_ (*t,x*) represents the cumulative distribution function (CDF) of the predicted binocular reaction times at a specific visual field location *x*. *F*_OS_(*t,x*) and *F*_OD_(*t,x*) are the cumulative distribution functions of the monocular reaction times for the OS and OD, respectively, measured at the same corresponding visual field location *x*. By calculating the model at each location *x*, the predicted binocular distribution reflects the statistical facilitation of independent monocular processes based on spatially localised visuomotor performance rather than global eye-level averages. This ensures that the model accounts for the regional variations in sensitivity and damage typically observed in glaucomatous visual fields. The expected binocular reaction time under the Race model was defined as the mean of the predicted binocular distribution derived from Eq. (2) for each corresponding visual field location (*x*). This point estimate reflects the probabilistic outcome of the stochastic ‘race’ between monocular channels, where the binocular response is triggered by whichever signal is faster on a given trial, and is not equivalent to simply selecting the faster of the two monocular means at that location.

As an alternative, a variance-weighted neural integration framework was implemented within the LATER model in which monocular inputs are combined according to their relative reliability at corresponding visual field locations [21–23]. Reliability is defined as the inverse of the variance of the promptness distribution, i.e., *w* = 1/*σ*^2^, such that responses with lower variability were assigned greater weight for each eye. The integrated binocular promptness (1/RT) is predicted as a weighted average:

3 $${P}_{{{OU}}}=\frac{{w}_{{{OS}}}{P}_{{{OS}}+}{w}_{{{OD}}}{P}_{{{OD}}}}{{{w}_{{{OS}}}+w}_{{{OD}}}}$$

where *P*_OS_ and *P*_OD_ denote monocular promptness (1/RT) at a given visual field location for the left and right eyes, respectively, and *w*_OS_ and *w*_OD_ represent the respective corresponding location-specific reliability weights. For each participant and visual field location, monocular reaction-time distributions were characterised using reciprobit analysis to obtain mean and standard deviation parameters, from which location-specific reliability weights were derived.

Binocular predictions were then computed separately for each visual field location under both the Race and variance-weighted models. For summary analyses, the resulting location-wise parameters and predicted reaction times were subsequently pooled across visual field locations (e.g., Fig. 3). This approach ensured that binocular integration and reliability were determined based on spatially localised visuomotor performance rather than global eye-level averages.

The Race and neural integration models were implemented to establish the theoretical boundaries of binocular interaction across the (glaucomatous) visual field. The Race model serves as a baseline for statistical facilitation, representing the theoretical lower bound of expected binocular benefit. In contrast, the neural integration model is rooted in Bayesian modelling, reflecting the theoretical upper bound of possible visuomotor performance. Together, these two models provide a computational framework to characterise the binocular summation patterns.

#### Bootstrap estimation and permutation testing of reaction times

**Fig. 2:**
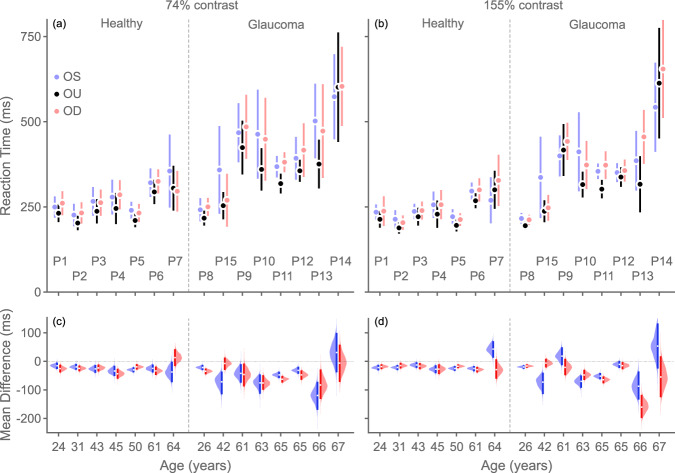
Comparison of monocular and binocular reaction times per individual target location. Mean reaction times across individual target locations per participant plotted as a function of age for low (74%; **a**) and high (155%; **b**) contrast targets. Healthy participants are shown on the left and glaucoma participants on the right, separated by a vertical dashed line. Data are colour-coded by viewing condition (left eye = OS, blue; right eye = OD, red; binocular = OU, black). Circles represent mean reaction times across target locations; vertical error bars denote the standard deviation across the means of all target locations. **c** and **d** Gardner–Altman estimation plots showing the mean differences between binocular and monocular reaction times for the same participants. Differences are expressed as OU–OS (blue) and OU–OD (red). The white notches indicate the mean difference, vertical bars show the 95% confidence intervals and the shaded patches represent the bootstrap distribution of the mean difference. Negative values indicate faster binocular responses relative to monocular, while positive values indicate slower binocular responses. If the 95% confidence intervals do not overlap with zero (dashed line), the two distributions differ significantly from each other.

**Fig. 3:**
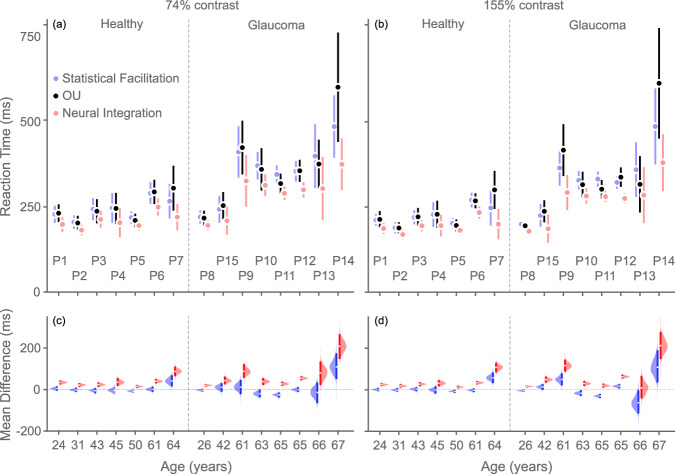
Comparison of observed binocular reaction times with computational model predictions. Mean reaction times per participant under binocular viewing (black) compared with model predictions from statistical facilitation (Race model; blue) and neural integration (red) for low (74%; **a**) and high (155%; **b**) contrast targets. **c** and **d** Gardner–Altman estimation plots showing the mean differences between empirical binocular data and model predictions (blue = statistical facilitation, red = neural integration). The layout, symbols, participant grouping and statistical conventions are identical to those detailed in Fig. 2. Negative values indicate observed binocular responses that were faster than the model predictions. OU both eyes.

**Fig. 4:**
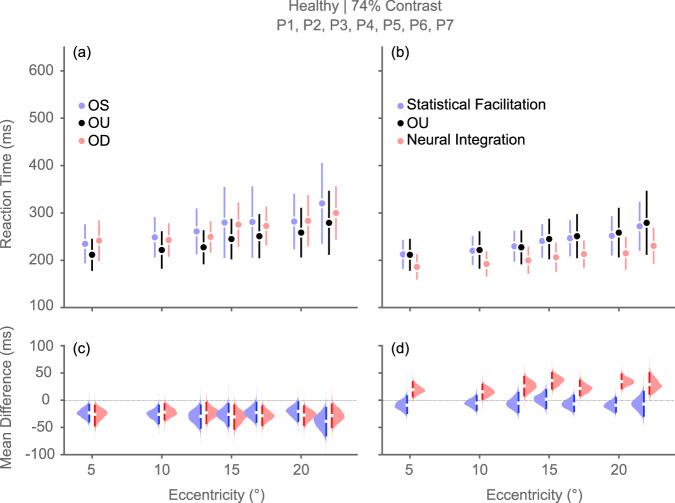
Binocular and monocular reaction times as a function of eccentricity in healthy participants. Mean reaction times (ms) plotted as a function of target eccentricity for binocular (black) and monocular (OS, blue; OD, red) viewing (**a**) and observed binocular viewing (black) compared with predictions from statistical facilitation (Race model; blue) and neural integration (red) (**b**). Gardner–Altman estimation plots showing the mean differences for the respective viewing condition comparisons (**c**) and model comparisons (**d**). The layout, colour-coding and statistical conventions, including the interpretation of bootstrap distributions, confidence intervals and significance, are identical to those detailed in Fig. 2. OD right eye, OS left eye, OU both eyes.

**Fig. 5:**
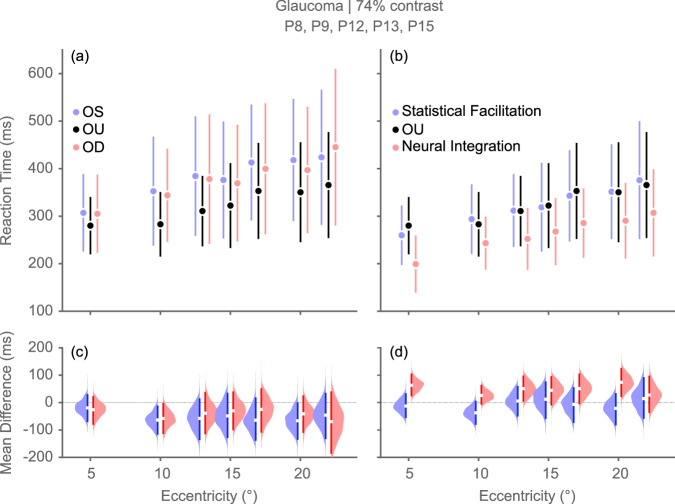
Binocular and monocular reaction times as a function of eccentricity in glaucoma participants (P8, P9, P12, P13, P15) exhibiting statistical facilitation. Same format as Fig. 4. OD, right eye; OS, left eye; OU, both eyes.

**Fig. 6:**
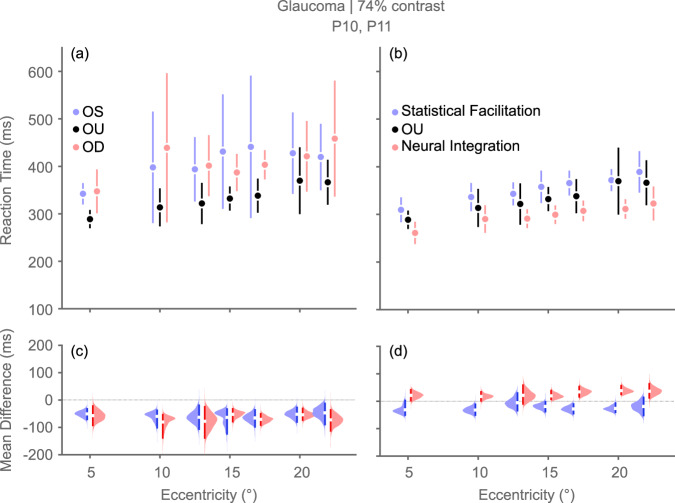
Binocular and monocular reaction times as a function of eccentricity in glaucoma participants (P10, P11) with intermediate behaviour reflecting a hybrid integration mode that surpasses pure statistical facilitation. Same format as Fig. 4. OD right eye; OS left eye; OU both eyes.

**Fig. 7:**
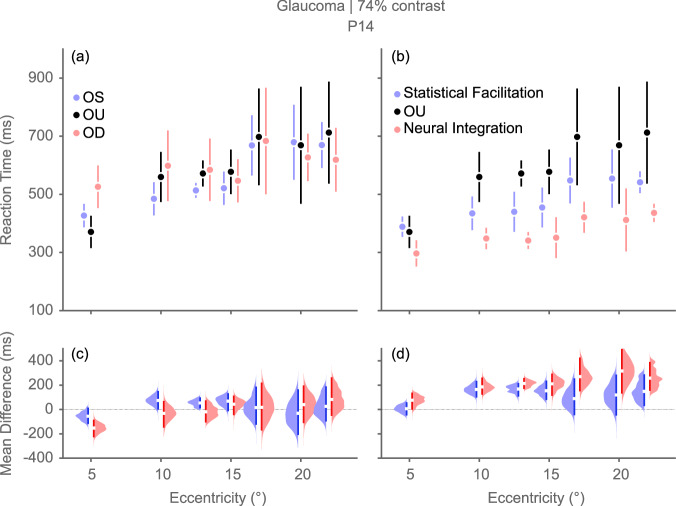
Binocular and monocular reaction times as a function of eccentricity in glaucoma participant P14 with disrupted binocular processing suggestive of an inhibitory interaction between the eyes. Same format as Fig. 4. OD right eye, OS left eye, OU both eyes.

Estimation statistics [44, 45] were used to compare reaction times (ms) across binocular and monocular viewing conditions (Fig. 2), between observed binocular reaction times and model predictions (Race and LATER; Fig. 3) and for within-eccentricity comparisons for viewing conditions and model predictions (Figs. 4–7).

A bootstrap procedure was employed that preserved both trial-level variability and participant-level structure. Within each of 1000 bootstrap iterations, trials were resampled with replacement separately for each participant and condition, and mean reaction times were computed. For each participant, the condition difference (e.g., binocular–monocular, i.e., OD, OS; binocular-model prediction, i.e., Race, LATER) was then calculated, from which the point estimate was reported, as well as the 95% confidence interval (CI) and margin of error (MoE), being the average length of the two CI arms.

Statistical significance was assessed using a permutation test on the original (non-bootstrapped) data. Specifically, the mean difference per participant was computed and the observed mean was compared to a null distribution generated by randomly flipping the sign across 10,000 permutations.

To improve readability, all results are reported as reaction times in ms instead of promptness values (see above and Eq. (1)). Results are reported as “difference ([lower 95% CI bound, upper 95% CI bound], MoE, *p*-value).” For binocular–monocular comparisons, a negative difference indicates faster reaction times under binocular viewing (binocular advantage). For binocular-model comparisons, a negative difference indicates observed binocular reaction times faster than the statistical facilitation/neural integration prediction. All statistical analyses were conducted at a significance level of *α* = 0.01.

#### Statistical Analysis with Linear Mixed-Effects Model

To investigate the effects of stimulus and demographic properties on reaction times, a linear mixed-effects model (LME) was implemented using MATLAB’s ‘*fitlme’* function. Promptness (1/RT) served as the dependent variable. To account for the hierarchical structure of the data and potential individual differences in sensitivity to testing conditions, a model was utilised that included both random intercepts and random slopes. The model was specified as:

4 $${{Prompt}}= \;	 {\beta }_{0}+{\beta }_{1}{{Group}}+{\beta }_{2}{{Age}}+{\beta }_{3}{{Contrast}} \\ 	 +{\beta }_{4}{{Eccentricity}}+{\beta }_{5}{{Eye}}+{\beta }_{6}({{Group}}\,x\,{{Contrast}}) \\ 	 +{\beta }_{7}({{Group}}\,x\,{{Eye}})+(1+{s}_{{{Contrast}}}+{s}_{{{Eccentricity}}}|{u}_{{{ID}}})+\varepsilon .$$

Promptness (Prompt), defined as the reciprocal of reaction time (1/RT) in inverse seconds (1/s), was modelled as a linear combination of fixed and random parameters. Fixed effects included group (categorical: healthy vs. glaucoma), age (years), contrast (categorical: 74% vs. 155%), eccentricity (degrees) and eye (categorical: left, right or both), with group × contrast and group × eye as interaction terms. The random-effects structure included a random intercept per participant (*u*_ID_) and random slopes for contrast (*s*_Contrast_) and eccentricity (*s*_Eccentricity_), allowing the effect of these within-participant variables to vary across individuals, as well as a residual error term, *ϵ*. The parameters in Eq. (4) are defined as follows: *β*_0_ is the population-level intercept, representing the mean promptness for the reference group (healthy participants viewing with the left eye at 74% contrast and 5° eccentricity), *β*_1_ through *β*_5_ are the fixed-effect coefficients for Clinical Group, Age, Contrast, Eccentricity and Eye, respectively, representing the estimated change in promptness per unit or category shift. *β*_6_ and *β*_7_ denote the interaction coefficients, quantifying how the effects of contrast and viewing condition differ between clinical groups. The participant-specific random intercept, *u*_ID_, represents the deviation of an individual participant’s baseline speed from the population intercept *β*_0_. Participant-specific random slopes, *s*_Contrast_ and *s*_Eccentricity_, represent individual deviations from fixed population effects of contrast (*β*_3_) and eccentricity (*β*_4_). To compare the measured binocular reaction times with the predictions of the statistical facilitation and neural integration models, a second LME model was implemented. This model was fitted to a combined dataset consisting of the empirically observed binocular responses, and the corresponding theoretical predictions were generated for each participant at every visual-field location. To account for the hierarchical nature of these comparisons and to maintain statistical consistency with the primary analysis, a structure was utilised that included both random intercepts and random slopes. The model was specified as:

5 $${{Prompt}}= 	 {\beta }_{0}+{\beta }_{1}{{Group}}+{\beta }_{2}{{Condition}}+{\beta }_{3}{{Eccentricity}}+{\beta }_{4}{{Age}} \\ 	 +{\beta }_{5}{{Contrast}}+(1 +{s}_{{{Contrast}}}+{s}_{{{Eccentricity}}}|{u}_{{{ID}}})+\varepsilon .$$

Fixed effects included group (categorical: healthy vs. glaucoma), age (years), contrast (categorical: 74% vs. 155%), condition (categorical: measured binocular responses, statistical facilitation prediction, neural integration prediction) and eccentricity (degrees). The random-effects structure consisted of a random intercept per participant (*u*_ID_) and random slopes for contrast (*s*_Contrast_) and eccentricity (*s*_Eccentricity_) grouped by participant. This configuration allows the model to account for individual heterogeneity in sensitivity to testing conditions while testing for systematic deviations between empirical data and theoretical models. The residual error is represented by *ϵ*. The parameters in Eq.(5) are defined as follows: *β*_0_ represents the population-level intercept, and *β*_1_ through *β*_5_ denote the fixed-effect coefficients for Clinical Group, Condition, Eccentricity, Age, and Contrast, respectively. In particular, *β*_2_ captures differences between empirically measured binocular responses and the theoretical predictions derived from statistical facilitation and neural integration models. The participant-specific random intercept, *u*_ID_, represents the deviation from the population intercept (*β*_0_), while random slopes, *s*_Contrast_ and *s*_Eccentricity_, represent individual deviations from the corresponding effects of contrast (*β*_5_) and eccentricity (*β*_3_), respectively.

For both models, random effects (*u, s*) are assumed to follow a multivariate normal distribution with mean zero and a variance-covariance matrix *D*, *B* ∼ *N*(0,*D*). The residual error term, ϵ, represents the unexplained per-observation noise or trial-by-trial fluctuations, assumed to be independently and identically distributed as ϵ∼*N*(0,σ^2^).

In the model-comparison analysis (Eq. (5)), eccentricity, age and contrast were included as fixed effects to account for known sources of variability in reaction time and to improve the precision of the statistical estimates. Although the theoretical Race and LATER model predictions are computed at the individual location level and inherently capture spatial performance, the inclusion of these covariates in the LME framework enables effective partitioning of residual variance in the empirical binocular observations. This facilitates a more sensitive evaluation of systematic deviations between empirical data and theoretical predictions. Models were fitted using maximum likelihood (ML). Model selection and the necessity of the random-slopes structure were assessed using the Akaike Information Criterion (AIC) and Bayesian Information Criterion (BIC), both of which supported the inclusion of the random-slopes model over a simpler random-intercept-only structure. Significance of fixed effects was determined using *F*-tests and *t*-tests, with *p*-values reported for all main and interaction terms.

### Ethics

All procedures performed in the study involving human participants adhered to the ethical standards of the Institutional Research Committee and the 1964 Helsinki Declaration and its later amendments or comparable ethical standards. The study was approved by the Medical Ethical Committee of the Erasmus University Medical Center, Rotterdam, The Netherlands (MEC-2022-0543) and the Institutional Review Board and Ethics Committee of Vision Research Foundation, Chennai, India (531-2023-P). Informed consent and consent to publish were obtained from all individual participants included in the study.

## Results

### Examples of Binocular Summation and Inhibition Across the Visual Field

Figure 1 depicts saccadic reaction times across the visual field as heat maps (first three columns) and reciprobit plots (fourth column) for monocular (OS, OD) and binocular (OU) viewing. The first row illustrates a healthy participant (Fig. 1a–d), while subsequent rows show examples of participants with glaucoma (Fig. 1e–l).

The heat maps display reaction times across 54 and 56 locations for monocular and binocular viewing, respectively, colour-coded from blue (faster) to red (slower). The reciprobit plots (Fig. 1d, h, l) display cumulative probability distributions of promptness pooled across all locations and repetitions, for each viewing condition. These plots provide a concise visualisation of eye-level differences by comparing the position of the binocular distribution with the monocular distributions [41].

For the healthy participant (P6; Fig. 1a–d), monocular responses to low-contrast stimuli were fast centrally (*R* ≤ 10°; 232–269 ms) and slower in the periphery (*R* > 15°; 307–382 ms), with a high proportion of goal-directed responses across locations. The binocular responses showed further improvement in central and mid-peripheral regions (10° < *R* ≤ 15°), consistent with binocular summation. The reciprobit plot confirmed this advantage, with the binocular distribution (black) shifted leftward relative to both monocular distributions (red and blue), indicating faster binocular responses.

In glaucoma participants, reaction times were generally slower and more variable, particularly at peripheral locations (*R* > 15°). Participant 14, with severe glaucoma, had the slowest responses overall, with reaction times of 342–495 ms centrally and up to 955 ms peripherally (Fig. 1e–g). At several test locations, the percentage of goal-directed saccades was reduced and for some extreme peripheral locations, no saccadic eye movement was made at all (black squares), consistent with total functional loss at these locations. Under binocular viewing (Fig. 1h), reaction time distributions (black) showed no clear advantage; they partially overlapped with monocular responses (red and blue) and, on average, were even slower and more variable.

Participant 15, with secondary glaucoma following blunt trauma to the left eye, exhibited severe impairment in the left visual field with a characteristic superior defect. Binocular reaction times (251 ± 74 ms; black) were only slightly faster than reaction times for the better right eye (264 ± 75 ms; red), offering only a weak reaction-time advantage. This suggests that binocular performance was essentially determined by the right eye, reflecting reduced binocular summation.

Occasionally, participants, e.g., P6 and P15, made goal-directed saccades (~1–2 saccades) toward blind-spot targets. Minor head misalignments during testing may have caused small position deviations. These locations were excluded from further analyses for all participants.

In summary, the healthy example participant demonstrated a clear binocular advantage, while advanced or asymmetric cases showed little benefit or evidence of binocular inhibition.

### Location-Based Analysis of Monocular and Binocular Reaction Times

In contrast to the reciprobit plots shown in Fig. 1d, h, l, which summarise reaction time mean and standard deviation pooled across the whole visual field, Fig. 2 presents data aggregated at the level of individual target locations. For each participant and viewing condition, mean reaction times (markers) per location and the standard deviation across the means of all locations (error bars) were calculated using the reciprobit analysis. This location-wise analysis enabled direct comparison of monocular and binocular reaction times at corresponding visual-field locations and yields a clearer representation of integration effects across the visual field. Estimation statistics were used to evaluate potential differences between reaction times in both monocular viewing conditions against binocular viewing (Fig. 2c and d).

For both low (Fig. 2a) and high (Fig. 2b) contrast targets, monocular reaction times of most healthy participants were well matched (smallest deviation 3 ms for P6 and largest deviation 11 ms for P1) except for P7 (60 ms), who presented with asymmetric visual acuity. Binocular reaction times of healthy participants were faster, on average, by 24 ms, indicative of binocular summation. P3 exhibited the smallest difference (155% WC, OS = –13 ms [–24, –3] ms, MoE = 10 ms, *p* < 0.001) and P4 exhibited the largest difference (74% WC, OD = –43 ms [–59, –27] ms, MoE = 16 ms, *p* < 0.001). Binocular reaction times of P7 showed right eye dominance for both the low contrast (13 ms [–17, 42] ms, MoE = 29 ms, *p* = 0.38) and the high contrast (–28 ms [–62, 6] ms, MoE = 34 ms, *p* = 0.02) conditions, likely reflecting interocular asymmetry in their visual acuity.

Compared to the sample of healthy participants, monocular reaction times of glaucoma participants were less well matched (the smallest deviation was 9 ms for P8 and the largest deviation was 89 ms for P15) and both monocular and binocular reaction times were more variable (compare the length of the error bars). Nevertheless, on average, binocular reaction times were faster for all glaucoma participants except for P14 (severe glaucoma) and P15 (unilateral, secondary glaucoma). Interestingly, the mean difference between monocular and binocular reaction times tended to be larger in the glaucoma group; ~42 ms (range: −13 to 112 ms). P8 exhibited the smallest difference (74% WC, OD = –21 ms [–31, –12] ms, MoE = 49 ms, *p* < 0.001) while P13 exhibited the largest difference (74% WC, OS = –118 ms [–169, –70] ms, MoE = 49 ms, *p* < 0.001). Regarding P14, binocular reaction times to targets presented at 74% contrast did not differ significantly from reaction times measured monocularly with the left eye (33 ms [–38, 98] ms, MoE = 68 ms, *p* = 0.59) or the right eye (–4 ms [–68, 62] ms, MoE = 65 ms, *p* = 0.39). The same was true for the 155% contrast targets (OS: 51 ms [–18, 132] ms, MoE = 75 ms, *p* = 0.0005; OD: 54 ms [–121, 22] ms, MoE = 72 ms, *p* = 0.01). For P15, binocular reaction times were equal to the non-affected right eye (74% WC: –8 ms [–31, 14] ms, MoE = 23 ms, *p* = 0.21) but faster than the affected left eye (74% WC: –73 ms [–114, –35] ms, MoE = 40 ms, *p* < 0.001).

In summary, healthy participants demonstrated robust binocular summation, whereas glaucoma participants showed greater variability, ranging from preserved summation to evidence of inhibition or monocular dominance.

For further statistical characterisation, reaction times were analysed using a linear mixed-effects model fitted to 4242 observations. The model utilised 10 fixed-effects coefficients and a random-effects structure with seven covariance parameters (including random slopes for contrast and eccentricity) and converged successfully with acceptable fit indices (AIC = 5764.5; BIC = 5872.5). Trial-by-trial fluctuations were the primary source of variance.

Glaucoma significantly reduced promptness (*β* = −0.94 1/s, *p* < 0.001), which corresponds with a slowing of reaction times by 33 ms compared to healthy controls. Promptness also decreased significantly with age (*β* = −0.028 1/s per year, *p* < 0.001), equivalent to a delay of ~1 ms per year. Responses were influenced systematically by stimulus properties. High target contrast significantly increased promptness (*β* = 0.33 1/s, *p* < 0.001), speeding responses by ~9 ms, whereas increasing eccentricity significantly slowed responses (*β* = −0.045 1/s per degree, *p* < 0.001), resulting in a delay of 1.3 ms per degree. Binocular viewing provided a robust advantage over monocular viewing (*β* = 0.39 1/s, *p* < 0.001), reducing reaction times by ~11 ms.

Notably, no significant interactions were found between group and contrast (*p* = 0.25) or group and eye (*p* = 0.89), indicating that the fundamental benefits of contrast and binocularity were qualitatively similar across both groups.

### Comparison of Observed Binocular Reaction Times with Model Predictions

To better understand the neural mechanisms underlying individual differences in binocular reaction times, observed data were compared with predictions from two computational frameworks, i.e., statistical facilitation (Race model) and neural integration based on Bayesian variance weighting (Fig. 3). Figure 3 follows the same format as Fig. 2 with binocular data being replotted in black and statistical facilitation and neural integration predictions shown in blue and red, respectively. In the mean difference panels (Fig. 3c, d), negative values indicate faster binocular responses than predicted, while positive values indicate slower responses.

In healthy participants, binocular reaction times were generally well accounted for by the statistical facilitation model. For six out of seven participants (P1–P6), 95% CIs for the differences overlapped with zero, indicating no significant deviation from statistical facilitation (Fig. 3c, d blue patches). In contrast, binocular responses consistently diverged from the neural integration model, which systematically underestimated observed reaction times (Fig. 3c, d red patches). This pattern suggests that binocular performance in normal vision is best explained by statistical facilitation of independent monocular signals, in line with previous studies [2, 3]. One healthy participant (P7) showed significantly slower binocular responses than predicted by either the statistical facilitation model (74% contrast: 41 [15,70] ms, MoE = 28 ms, *p* < 0.001; 155% contrast: 58 [31,84] ms, MoE = 26 ms, *p* < 0.001) or the neural integration model (74% contrast: 87 [63, 112] ms, MoE = 25 ms, *p* < 0.001; 155% contrast: 108 [84, 130] ms, MoE = 23 ms, *p* < 0.001), likely reflecting interocular asymmetry in their visual acuity.

The glaucoma group showed more heterogeneous behaviour. At low contrast (74%), most participants also conformed to statistical facilitation predictions, but distinct subgroups emerged (Fig. 3c). For the largest group (P8, P9, P12, P13, P15) binocular reaction times did not differ significantly from the statistical facilitation predictions, with the largest effect size observed for P13 (–14 [–61, 34] ms, MoE = 47 ms, *p* = 0.54) and the smallest for P12 (–1 [–13, 11] ms, MoE = 12 ms, *p* = 0.73). The second response type, seen in P14, was characterised by binocular reaction times being significantly slower than the statistical facilitation predictions (107 [48 165] ms, MoE = 59 ms, *p* = 0.0001). In the last group, binocular reaction times were faster than predicted by statistical facilitation, suggesting the presence of some form of neural integration. This was especially clear in P11 (–26 [–38, –16] ms, MoE = 11 ms, *p* < 0.001) and less so in P10 (–20 ms [–38, –1] ms, MoE = 18 ms, *p* = 0.02).

At high contrast (155%), binocular responses again diverged strongly from the neural integration model, but group-level conformity to statistical facilitation was reduced (Fig. 3d). Three participants showed significantly slower binocular responses than predicted. This could be seen for P14 (104 [32, 175] ms, MoE = 72 ms, *p* = 0.001), P9 (49 [20,76] ms, MoE = 28 ms, *p* < 0.001) and P12 (17 [7,27] ms, MoE = 10 ms, *p* = 0.0006), suggesting possible inhibitory interactions or monocular dominance. However, two glaucoma participants showed binocular responses faster than statistical facilitation predictions. This was again the case for P10 (–17 [–31, –3] ms, MoE = 14 ms, *p* = 0.0005) and P11 (–31 [–41 –19] ms, MoE = 11 ms, *p* < 0.001). Participant 13 showed unexpected behaviour, in particular, binocular reaction times appeared slightly faster than the statistical facilitation model predictions (–64 ms [–117, –0.7] ms, MoE = 58 ms, *p* = 0.03) and seemed similar to the neural integration model (6 [–43 66] ms, MoE = 55 ms, *p* = 0.09).

To evaluate how measured binocular reaction times compared with model predictions, a linear mixed-effects model (LMEM) was fitted to 4242 observations. The model utilised seven fixed-effects coefficients and seven random-effects covariance parameters (including random slopes for contrast and eccentricity), which provided a good fit (AIC = 7972.2; BIC = 8061.2) compared to a random-intercept-only structure.

Glaucoma significantly reduced promptness compared with healthy controls (*β* = −0.79 1/s, *p* < 0.001), representing a 24 ms slowing of reaction times. Promptness also decreased with age (*β* = −0.027 1/s, *p* < 0.001), equivalent to ~1 ms per year, and with eccentricity (*β* = −0.051 1/s, *p* < 0.001), equivalent to 1.6 ms per degree. High target contrast significantly increased promptness (*β* = 0.30 1/s, *p* < 0.001), equivalent to a speed-up of 7 ms. Regarding model performance, measured binocular reaction times did not differ significantly from the statistical facilitation (Race model) prediction (*β* = 0.029 1/s, *p* = 0.20). However, the neural integration model systematically and significantly overestimated promptness (*β* = 0.71 1/s, *p* < 0.001), predicting responses typically 17 ms faster than those measured empirically.

To evaluate the absolute accuracy of the theoretical models at the individual location level, the root mean square error (RMSE) was calculated between empirical binocular observations and model predictions for each group. For the healthy cohort, the Race model demonstrated high fidelity with a typical deviation of only 29 ms, whereas the neural integration model showed a larger error of 51 ms. In the glaucoma cohort, while absolute error increased due to the higher variability inherent in affected visual fields, the Race model remained considerably more accurate (76 ms) than the neural integration model (115 ms). These descriptive metrics complement the LMEM analysis (Eq. (5)), confirming that the statistical facilitation mechanism (Race model) not only aligns with group-level performance but also provides superior point-by-point precision across both healthy and glaucomatous participants.

In summary, the comparison between binocular reaction times and model predictions showed that observed binocular responses were overall more aligned with statistical facilitation, while predictions from neural summation exceeded the measured facilitation.

### Eccentricity-Dependent Analysis of Binocular Summation

The model-based comparisons in the previous section revealed that healthy participants exhibited binocular performance consistent with the statistical facilitation model. In contrast, the glaucoma group showed considerable heterogeneity, ranging from conformity with statistical facilitation to partial facilitation or inhibition. To further understand whether these distinct integration modes were spatially dependent, binocular reaction times were examined as a function of eccentricity and compared with computational model predictions (Figs. 4–7).

In healthy participants (Fig. 4a, c), binocular reaction times increased progressively with eccentricity from ~211 ms at 5° to 279 ms at 22°, corresponding to an overall increase of about 68 ms. Across all eccentricities, measured binocular reaction times closely followed the statistical facilitation prediction, with deviations remaining within ±10–12 ms and not reaching statistical significance (Fig. 4b, d). The model accurately captured the magnitude of binocular facilitation at both central and peripheral locations, indicating stable statistical facilitation across the field. In contrast, the neural integration model systematically overestimated binocular facilitation, predicting reaction times that were 20–34 ms faster than those measured, particularly at mid-peripheral and peripheral eccentricities (15°–20°).

In glaucoma participants (P8, P9, P12, P13, P15) exhibiting preserved binocular summation (Fig. 5a–d), binocular reaction times increased systematically with eccentricity, on average from 280 ms at 5° to 365 ms at 22°, corresponding to an overall eccentricity-related delay of about 85 ms. This increase closely mirrored the monocular trend. Measured binocular reaction times remained well aligned with statistical-facilitation predictions, with deviations typically in the range of ±20 ms and not reaching statistical significance across eccentricities. In contrast, the neural integration model systematically overestimated binocular facilitation, predicting reaction times that, on average, were 40–70 ms faster than those observed, particularly beyond 15° eccentricity.

In two participants (P10, P11), binocular reaction times were faster than predicted by statistical facilitation but slower than those expected under neural integration (Fig. 6a–d). Reaction times increased from ~288 ms at 5° to 366 ms at 22°, paralleling the typical eccentricity-dependent slowing observed in monocular viewing. Across eccentricities, measured binocular responses exceeded the statistical facilitation predictions by 20–30 ms for several central and mid-peripheral locations, indicating a moderate enhancement of visuomotor speed beyond probability summation. However, the neural integration model consistently overestimated binocular reaction times, predicting reaction times 15–50 ms faster than those measured, especially in the mid-periphery (15°–20°).

One participant with severe glaucoma (P14) exhibited binocular inhibition, characterised by reaction times that were consistently slower than both monocular responses and statistical facilitation predictions (Fig. 7a–d). Binocular reaction times increased from 370 ms at 5° to 713 ms at 22°, corresponding to a total eccentricity-dependent delay of over 343 ms. Except for 5° eccentricity, measured binocular responses were 100–160 ms slower than the statistical facilitation model and significantly exceeded its confidence bounds, indicating a robust inhibitory interaction between the eyes. The neural integration model also failed to predict the measured binocular reaction times satisfactorily, overestimating binocular reaction times by more than 200 ms relative to the measured data. The inhibition was spatially uniform across eccentricities, suggesting a global disruption of binocular processing rather than a localised effect.

Across all groups, reaction times increased with eccentricity, but the mode of binocular integration, whether probability-based, partially facilitated or inhibitory, remained stable across spatial locations. No strong evidence of eccentricity-specific modulation was observed. Furthermore, qualitatively similar patterns were observed at high contrast (155%), with the same group-specific differences maintained across eccentricities, although absolute reaction times were faster overall. These findings indicate that while eccentricity influences overall reaction-time speed, the underlying integration mechanism remains consistent across the visual field.

## Discussion

The present study provides the first systematic characterisation of binocular reaction times in glaucoma using EMP. By extending reaction-time analysis to binocular viewing, the findings show that while healthy participants consistently exhibited a clear binocular advantage, patients with glaucoma demonstrated marked heterogeneity in their binocular integration.

### Binocular Reaction Times across Healthy and Glaucomatous Visual Fields

In healthy participants, binocular reaction times were on average 20 ms faster than monocular responses, closely matching predictions from statistical facilitation and diverging from a neural integration model based on variance-weighting, which systematically predicted faster binocular reaction times. This finding is consistent with earlier psychophysical studies, which demonstrated that binocular performance in normal vision can be explained largely by the statistical facilitation of independent monocular signals [2, 3, 15]. This pattern demonstrates that, in normal vision, binocular performance reflects statistical facilitation of independent monocular inputs rather than full reliability-weighted integration.

In contrast, participants with glaucoma displayed more variable patterns. While the majority in the current sample (P8, P9, P12, P13, P15) exhibited preserved binocular speed-up consistent with statistical facilitation (~40 ms faster), others showed deviations from this pattern. One participant with severe glaucoma (P14) demonstrated binocular inhibition, with binocular responses slower than monocular performance, consistent with impaired binocular summation reported in glaucoma under conditions of marked interocular asymmetry [25, 37]. This pattern indicates a breakdown of the probabilistic combination of monocular signals, resulting in binocular responses that are slower than expected from independent processing. In contrast, one participant with secondary glaucoma in their left eye (P15) exhibited responses consistent with statistical facilitation but effectively dominated by the better eye, suggesting that the contribution of the weaker eye was minimal. Such better-eye dominance has been described in conditions of asymmetric visual function [38, 46]. This reflects an adaptive reliance on the less affected eye rather than balanced binocular integration. The absence of binocular summation in such cases is in line with previous reports linking interocular asymmetry with reduced stereopsis and contrast sensitivity [47, 48]. More broadly, interocular asymmetries (for example, reduced acuity or subclinical media opacities) [25, 46] can attenuate binocular summation and have been associated with impaired driving performance and postural balance [49, 50].

The present sample also contained two glaucoma participants (P10, P11) whose binocular responses were faster than statistical facilitation predictions, but remained slower than those expected under neural integration based on variability weighting, suggesting partial facilitation that does not reach the level of optimal cue combination. In healthy vision, binocular summation can, under certain conditions, exceed the level predicted by statistical facilitation, reflecting genuine neural interaction between the eyes [18]. Such “neural summation” is most reliably observed for weak or near-threshold stimuli [3, 32, 51, 52]. The intermediate behaviour observed in these participants suggests a hybrid integration mode that surpasses pure statistical facilitation but falls short of full reliability-weighted neural integration, indicating that binocular enhancement may be limited by the reduced reliability of visual input in glaucoma. Although neural summation can exceed statistical facilitation under conditions of weak stimulation, the present findings indicate that binocular performance in glaucoma more commonly reflects statistical facilitation or partial integration rather than full neural summation. Collectively, these observations illustrate distinct manifestations of altered binocular processing (binocular inhibition, better-eye dominance and partial facilitation) associated with asymmetric or advanced disease, and underscore the heterogeneity of binocular integration in glaucoma.

An important question arising from these findings is whether these integration modes vary across visual field locations or remain spatially consistent. To address this, reaction times were examined as a function of eccentricity. This analysis confirmed that binocular integration patterns were spatially stable across the visual field. As reported earlier, reaction times increased from central to peripheral locations for all groups [53], but the mode of binocular summation, whether probability-based, neural summation or inhibitory, remained consistent across eccentricities irrespective of contrast.

Importantly, although reaction times increased systematically with eccentricity (~1.3–1.6 ms per degree), monocular performance trajectories were frequently non-parallel, particularly in glaucoma. Due to the localised nature of glaucomatous damage, the relative efficiency of the two eyes varied across visual field locations. An eye providing faster responses centrally could become slower in peripheral regions, depending on regional functional loss. This spatial variability indicates that while the mechanism of integration is spatially consistent, the relative contribution of each eye to the binocular response is inherently location-dependent. Consequently, binocular performance in glaucoma cannot be characterised by a single global functional classification, such as the ‘better’ or ‘worse’ eye, as an eye’s relative efficiency may flip depending on the local functional status of the tested region.

### Modelling Binocular Reaction Times

Computational model predictions were compared with observed binocular reaction times. A practical motivation for modelling binocular reaction times is the possibility of predicting everyday binocular performance from monocular reaction-time data, similar to binocular Humphrey Esterman data based on monocular Humphrey fields [30–32]. If reliable predictions were feasible, then this would reduce the testing burden substantially while offering a more ecologically valid estimate of visuomotor function under natural viewing, where patients use both eyes simultaneously. However, the present findings indicate that monocular reaction times alone are insufficient to determine whether a given patient will exhibit statistical facilitation, partial neural summation, better-eye dominance or binocular inhibition. Prior work suggests that interocular asymmetry is an important determinant of binocular interactions [25, 37, 38] and may therefore be a key factor for predicting whether binocular combination will enhance or impair performance. Yet the present dataset does not reveal a straightforward threshold or rule linking the magnitude or form of monocular asymmetry to the resulting binocular summation pattern. Developing predictive frameworks will likely require integrating monocular reaction-time distributions with structural or reliability-based metrics (e.g., contrast sensitivity, ganglion-cell integrity or visual-field concordance) known to modulate binocular integration [22, 23, 28].

Alternatively, the 4th-root summation rule, a Minkowski-type framework (*k* = 4), that is traditionally applied to model binocular contrast detection thresholds, may be an alternative to the current neural integration approach [6, 10]. While this rule is well-established in the threshold domain, reaction-time paradigms conventionally rely on the Race model, which describes a stochastic “MAX-rule” competition between independent monocular signals [17, 18]. A fundamental distinction exists between the static nature of a fixed summation rule and the dynamic framework of the present neural integration model. The latter utilises reliability-based weighting (*w* = 1/*σ*^2^) to allow the relative contribution of each eye to shift adaptively based on localised glaucomatous damage [22, 23]. Nevertheless, the 4th-root model may be theoretically superior in specific circumstances, particularly when characterising binocular performance for weak or near-threshold stimuli where active neural summation results in a gain that exceeds the statistical facilitation predicted by an independent race significantly [3, 32, 51]. Future work combining monocular EMP, structural biomarkers and computational modelling is necessary to determine when binocular facilitation, inhibition or intermediate modes of integration can be anticipated from clinically accessible measures.

### Methodological Considerations

A key contribution of this study is the methodological approach. To enable direct, location-wise comparisons across the visual field, monocular (left, right) and binocular reaction times were measured across the visual field with both low and high contrast targets, using multiple repetitions per condition. This resulted in a detailed but time-intensive protocol and therefore, the study was conducted in a small cohort of seven healthy participants and eight participants with glaucoma spanning a range of disease severities. The emphasis was not on clinical generalisation but on providing an initial, high-resolution characterisation of binocular summation in glaucoma based on reaction times and establishing a methodological framework for future studies.

While the cohort size was small, the detailed nature of the data provides a proof-of-concept for the application of binocular EMP in probing underlying mechanisms of integration. Larger studies will be required to determine variations in binocular reaction times across glaucoma subtypes, disease stages and their associations with structural measures such as Optical Coherence Tomography [54, 55]. Furthermore, linking binocular reaction times to functional outcomes, including simulated driving [56, 57] or mobility tasks [58–60], will be an important step toward establishing their clinical utility.

### Clinical Relevance

The reduced or inconsistent binocular advantage that can be observed in glaucoma has real-world consequences. Binocular vision typically provides perceptual and motor benefits in healthy individuals, particularly for tasks requiring rapid visual-motor coordination [4]. However, in asymmetric glaucoma, this advantage appears diminished or absent, potentially impairing performance in everyday activities such as driving, navigating obstacles, hazard detection or functioning under low-visibility conditions [61–65]. Importantly, functional impairments and reduced quality of life can emerge before standard perimetric tests detect significant field loss [66].

From a diagnostic perspective, binocular reaction-time measurements complement traditional tests by providing additional insights into functional vision. Reaction times provide a particularly sensitive metric in this respect. Unlike threshold-based measures, they capture the dynamics of sensory processing and motor initiation, thereby offering a complementary index of functional visual capacity. Reduced or absent binocular summation could serve as a behavioural marker in asymmetric glaucoma. Therefore, incorporating binocular reaction-time tasks into clinical evaluations may improve functional validity by aligning assessments with real-world demands and assessing the impact of the disease. These approaches could help detect subtle functional impairments that remain undetected by conventional clinical evaluations [32].

## Conclusions

The present findings demonstrate that healthy participants show robust binocular summation consistent with statistical facilitation, whereas glaucoma is associated with diverse integration outcomes, ranging from preserved summation to inhibition. Reaction times offer a sensitive measure of visual processing efficiency, while binocular EMP provides a useful methodological tool to investigate functional consequences of glaucomatous damage.

## Data Availability

The dataset is available in the figshare repository, https://figshare.com/s/2ff418db33e491a8e6cb.

## Code availability

The analysis code is available in the figshare repository, https://figshare.com/s/2ff418db33e491a8e6cb.
